# Author Correction: Inhibitory mechanism of reveromycin A at the tRNA binding site of a class I synthetase

**DOI:** 10.1038/s41467-021-22942-2

**Published:** 2021-04-29

**Authors:** Bingyi Chen, Siting Luo, Songxuan Zhang, Yingchen Ju, Qiong Gu, Jun Xu, Xiang-Lei Yang, Huihao Zhou

**Affiliations:** 1 Guangdong Provincial Key Laboratory of Chiral Molecule and Drug Discovery, School of Pharmaceutical Sciences, Sun Yat-sen University, Guangzhou, 510006 China; 2 Research Center for Drug Discovery, School of Pharmaceutical Sciences, Sun Yat-sen University, Guangzhou, 510006 China; 3 Department of Molecular Medicine, Scripps Research Institute, La Jolla, CA 92037 USA

**Keywords:** Enzymes, X-ray crystallography, Mechanism of action, tRNAs

Correction to: *Nature Communications* 10.1038/s41467-021-21902-0, published online 12 March 2021.

The original version of this Article contained an error in the author affiliations.

Affiliation number 1 incorrectly read ‘Guangdong Provincial Key Laboratory of Chiral Molecule and Drug Discovery, Sun Yat-sen University, Guangzhou 510006, China’.

This has now been corrected in both the PDF and HTML versions of the Article to ‘Guangdong Provincial Key Laboratory of Chiral Molecule and Drug Discovery, School of Pharmaceutical Sciences, Sun Yat-sen University, Guangzhou 510006, China’.

