# Supplementary material for: Inhibitory mechanism of reveromycin A at the tRNA binding site of a class I synthetase
Source: Nat Commun. 2021 Mar 12;12:1616. doi: 10.1038/s41467-021-21902-0 (PMC7955072; doi:10.1038/s41467-021-21902-0)
Supplement: Supplementary file 1 — Supplementary Information [file 41467_2021_21902_MOESM1_ESM.pdf]

Supplementary Information for

Inhibitory mechanism of reveromycin A at the tRNA binding site of a class I synthetase

Bingyi Chen[1,2], Siting Luo[1,2], Songxuan Zhang[1,2], Yingchen Ju[1,2], Qiong Gu[2], Jun Xu[2], Xiang-Lei Yang[3], Huihao Zhou[1,2,*]

[1]Guangdong Provincial Key Laboratory of Chiral Molecule and Drug Discovery, and [2]Research Center for Drug Discovery, School of Pharmaceutical Sciences, Sun Yat-sen University, Guangzhou 510006, China. [3]Department of Molecular Medicine, Scripps Research Institute, La Jolla, CA 92037, USA.

[*]Corresponding authors: zhuihao@mail.sysu.edu.cn (H.Z.)

The supplementary material includes:

- Supplementary Figures 1-12
- Supplementary Tables 1-4

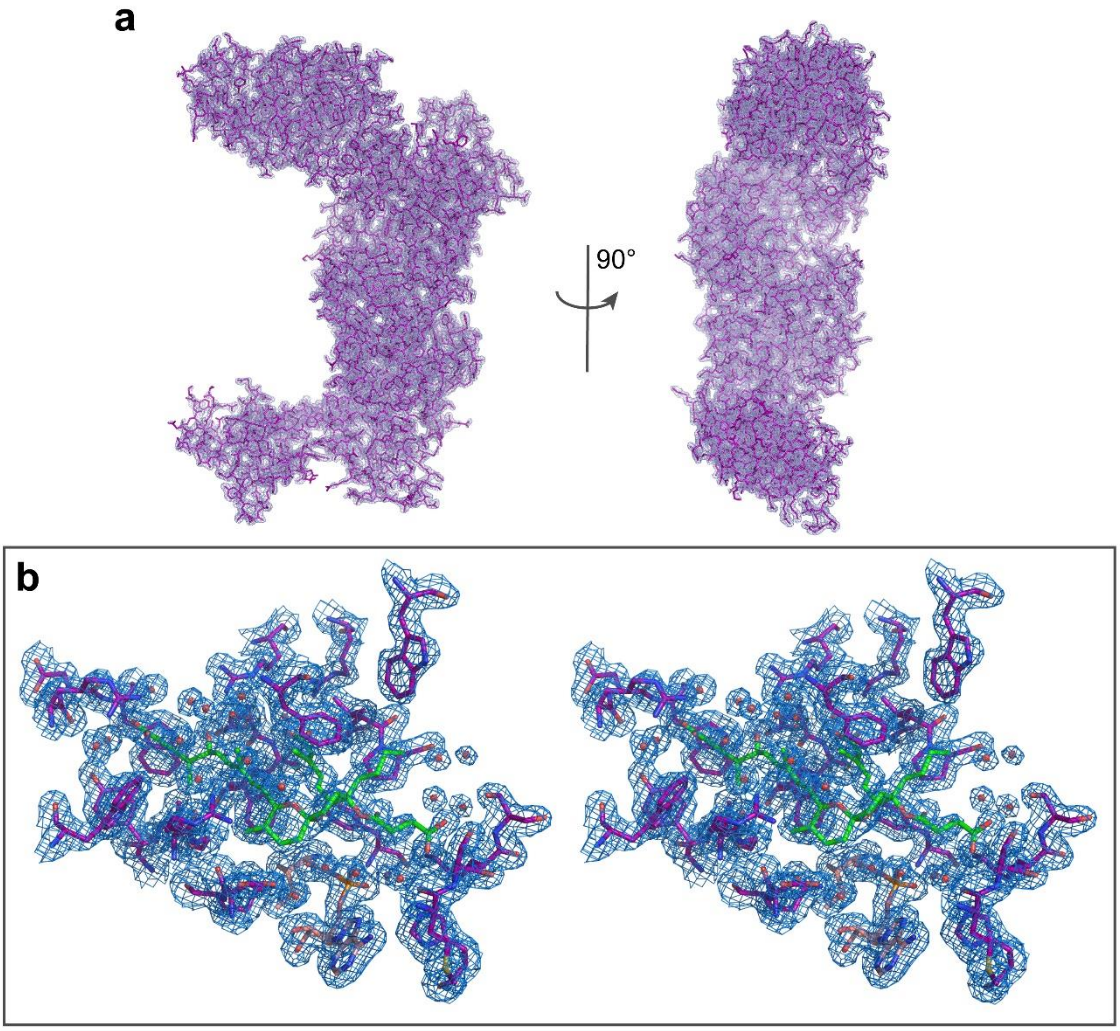

Supplementary Figure 1 | Electron density map of the *Sc*IleRS·RM-A·Ile-AMP ternary complex structure. a, The 2Fo-Fc electron density map of the protein chain. **b**, The stereo view of the 2Fo-Fc electron density map of the RM-A binding site in *Sc*IleRS. The electron density map is shown as mesh. RM-A and Ile-AMP molecules are shown as green and salmon sticks respectively. Protein chain is shown as sticks in purple. All maps were contoured at 1.0 σ.

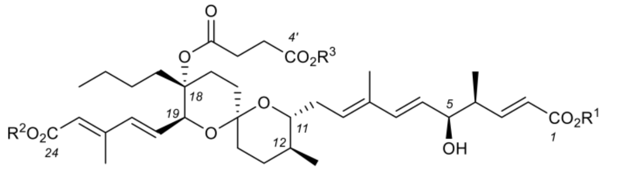

1: $R^1 = R^2 = R^3 = H$, RM-A
2: $R^1 = Me$, $R^2 = R^3 = H$
3: $R^2 = Me$, $R^1 = R^3 = H$
4: $R^3 = Me$, $R^1 = R^2 = H$

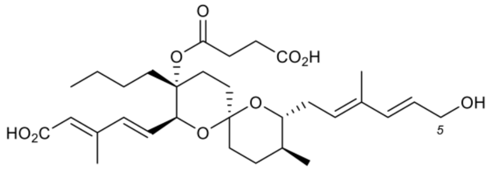

5

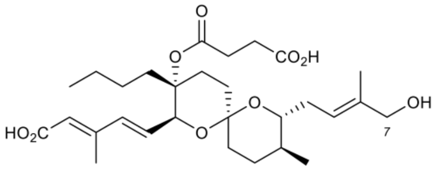

6

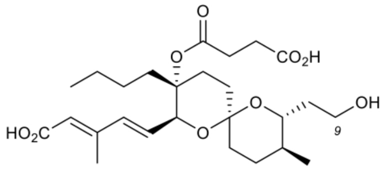

7

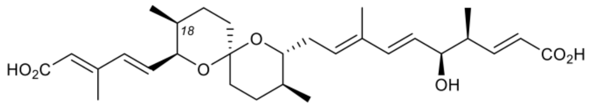

SF-A

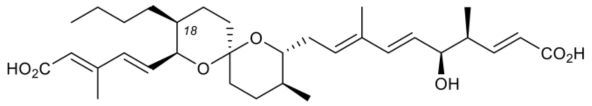

RM-T

	RM-A	**2**	**3**	**4**	**5**	**6**	**7**	SF-A	RM-T
IC_{50} on IleRS activity (nM)	4.5	300	>1000	417	996	>1000	>1000	>1000	0.4

Note: The activities of RM-A, **2-7** and SF-A were reported by Shimizu et al. [1].
The activity of RM-T was reported by Takahashi et al. [2].

Supplementary Figure 2 | The chemical structures of RM-A and its analogues, and their inhibitory activities against the aminoacylation reaction of IleRS.

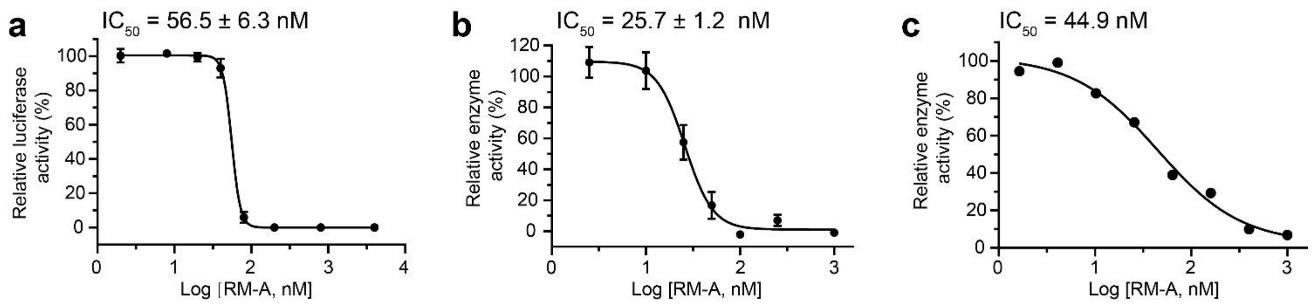

Supplementary Figure 3 | RM-A activity. a, Inhibition to in vitro translation. Data are shown as mean ± SD (n = 3 independent experiments). **b**, Inhibition to the aminoacylation activity of *Sc*IleRS. Data are shown as mean ± SD (n = 3 independent experiments). **c**, Inhibition to the pre-transfer editing activity of *Sc*IleRS. Data are shown as mean (n = 2 independent experiments). Source data are provided as a Source Data file.

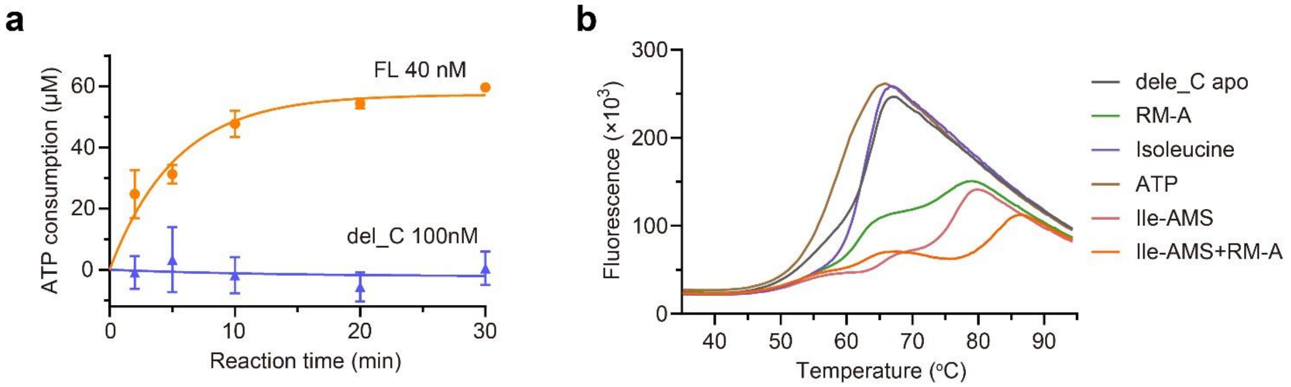

Supplementary Figure 4 | ATP consumption assay and thermal shift assay of C-terminal truncated *Sc*IleRS. a, As shown in ATP consumption assay, the C-terminal truncated *Sc*IleRS (del_C, residues 1-984) lost the aminoacylation activity, while full-length *Sc*IleRS (FL) is active. Data are shown as mean ± SD (n = 3 independent experiments). Source data are provided as a Source Data file. **b**, The thermal melting curves of the C-terminal truncated *Sc*IleRS in the presence of different ligands.

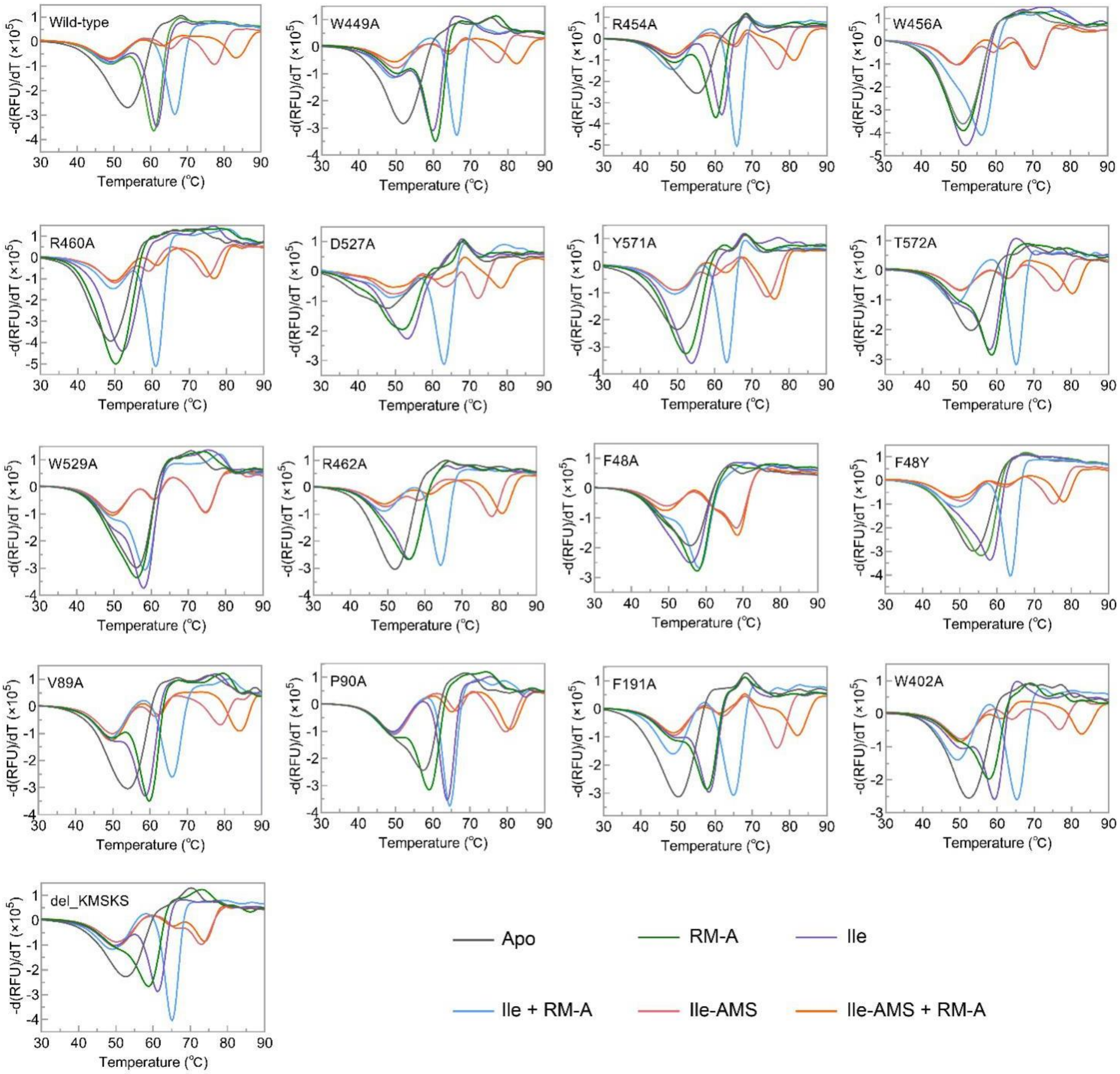

Supplementary Figure 5 | Thermal melting curves of *Sc*IleRS and its variants in the presence of different ligands.

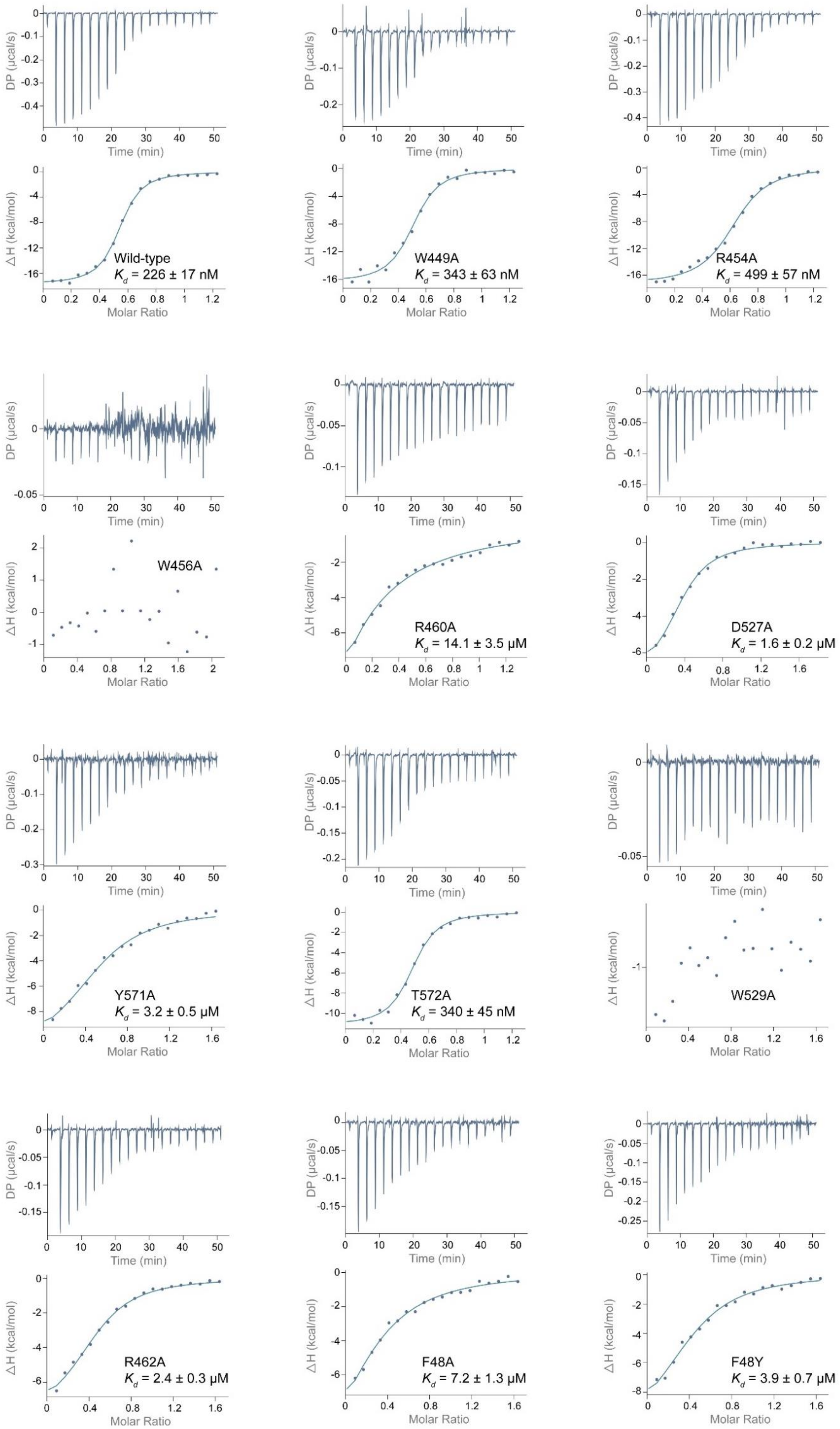

(continued on next page)

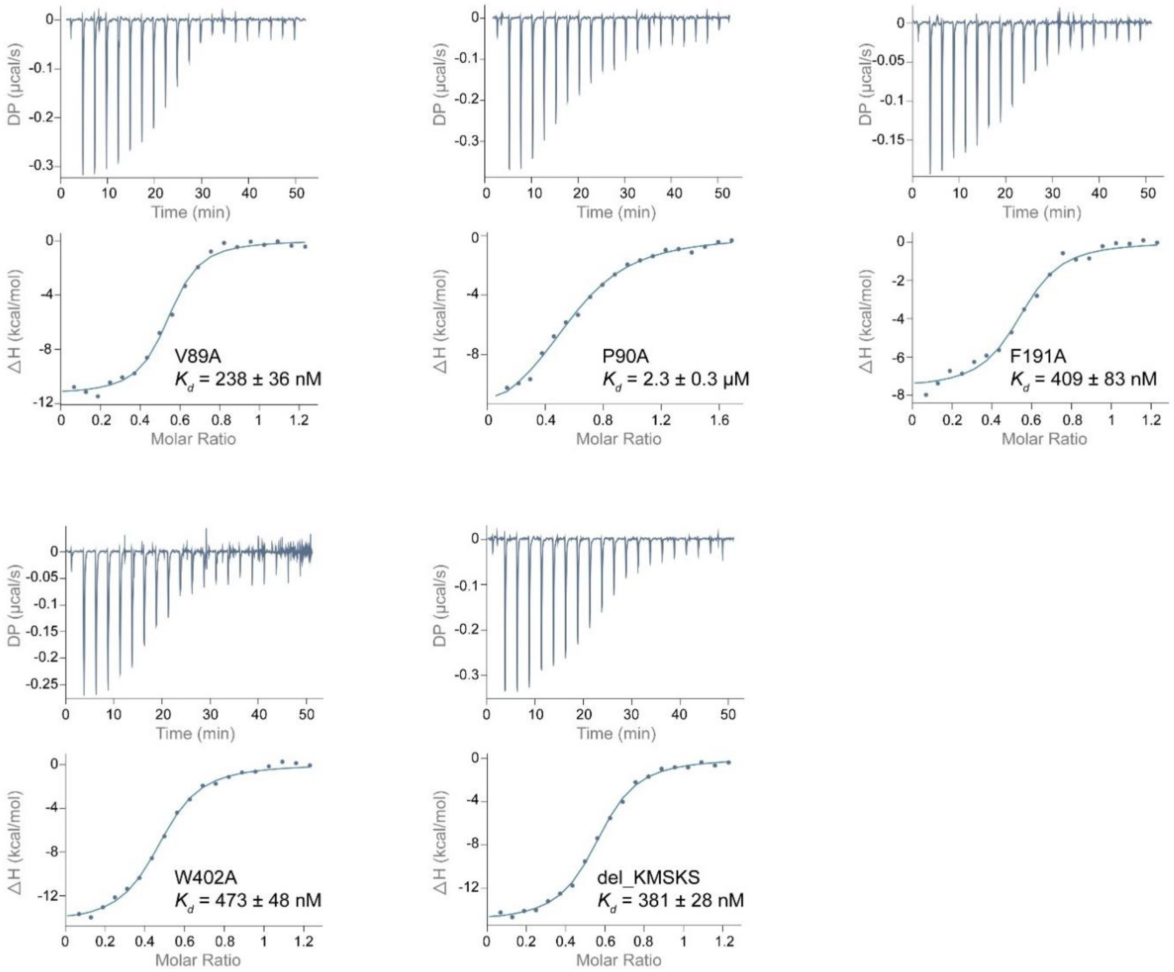

Supplementary Figure 6 | ITC titrations of RM-A to *Sc*IleRS and its variants.

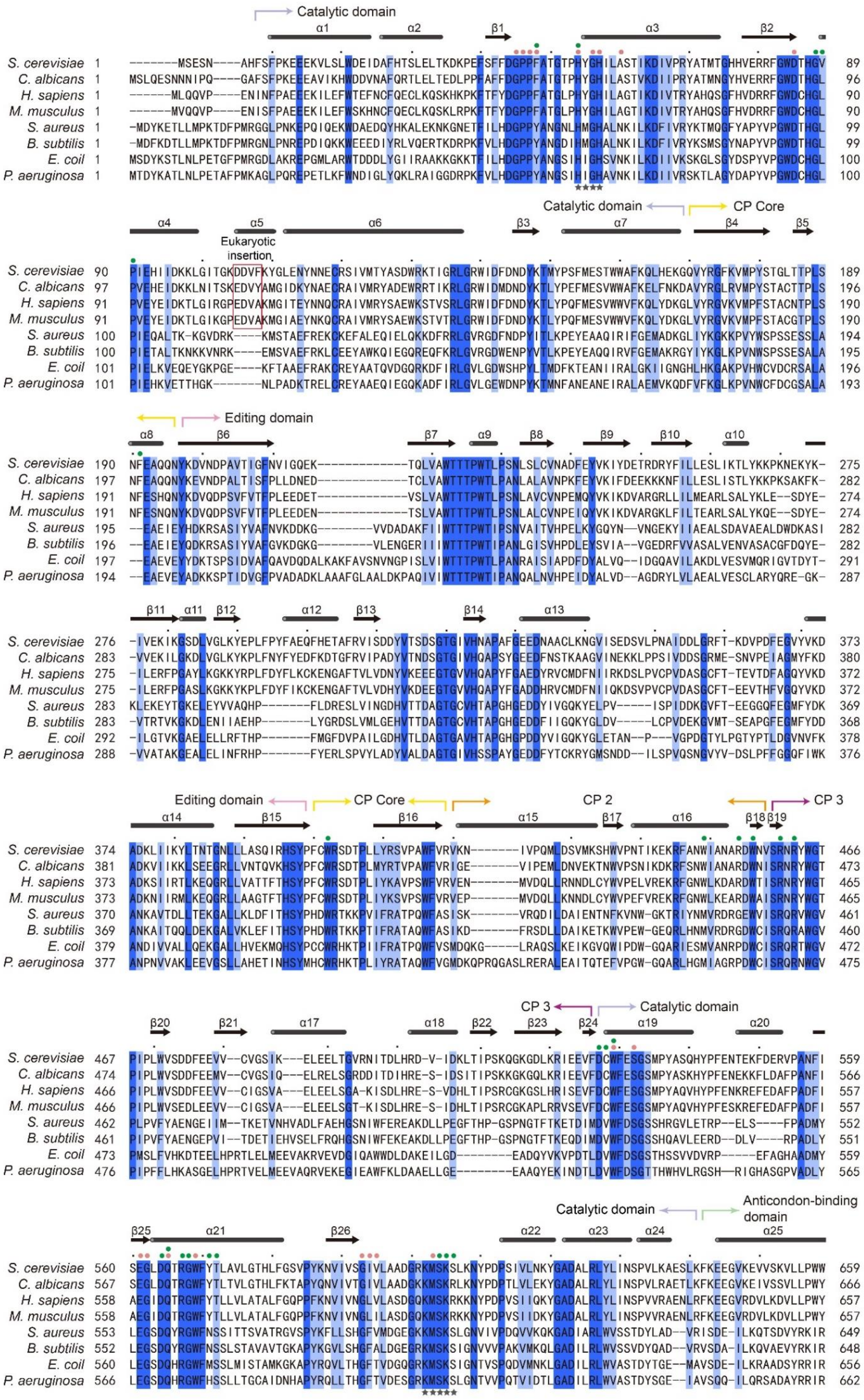

(continued on next page)

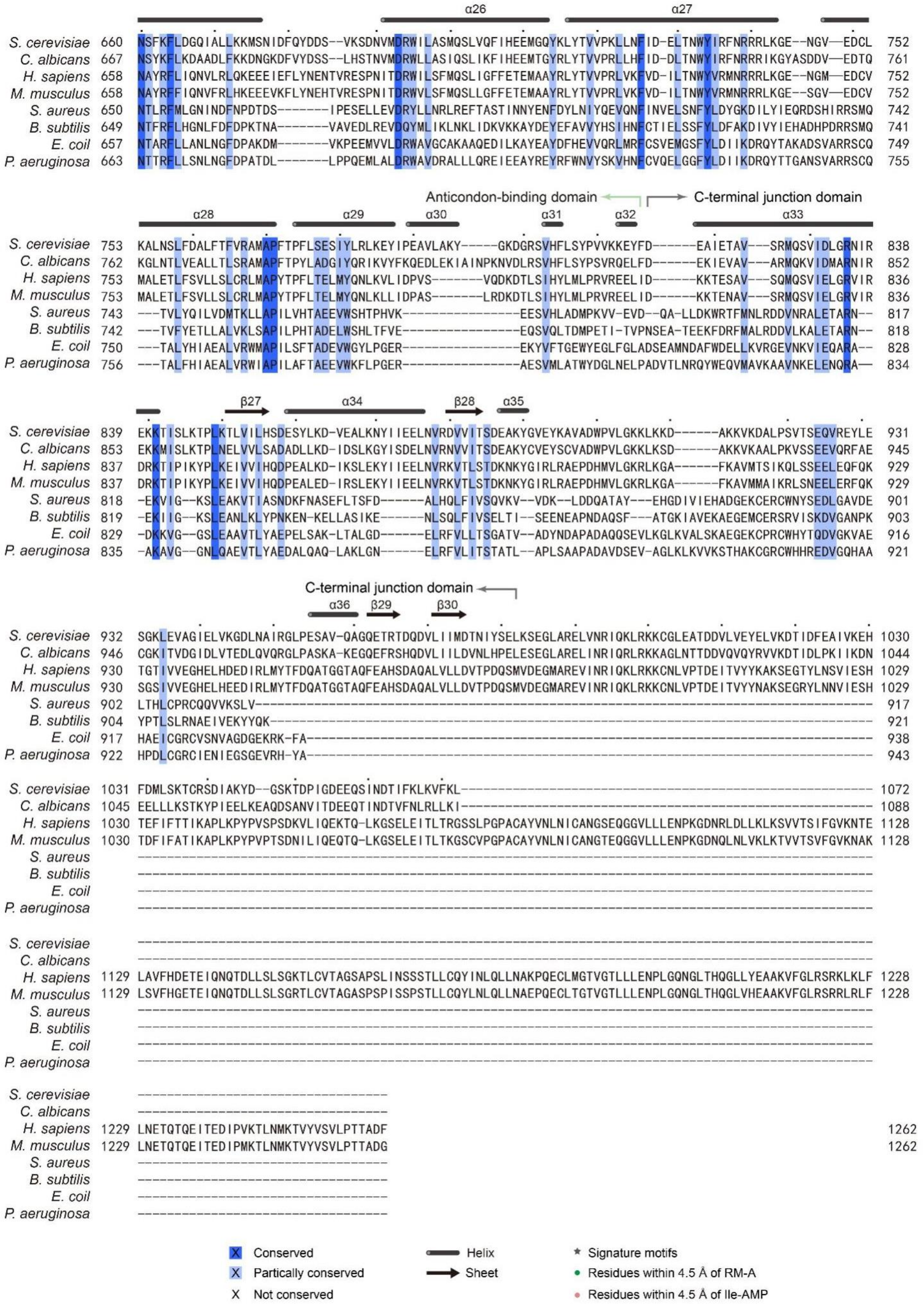

Supplementary Figure 7 | Structure-based sequence alignments of IleRS orthologues. The protein sequences of eukaryotic cytoplasmic IleRSs from *S. cerevisiae*, *C. albicans*, *H. sapiens* and *M. musculus* and bacterial IleRSs from *S. aureus*, *B. subtilis*, *E. coil*, and *P. aeruginosa* were aligned using Clustal Omega program[3]. The secondary structures corresponding to *Sc*IleRS are displayed above the sequences.

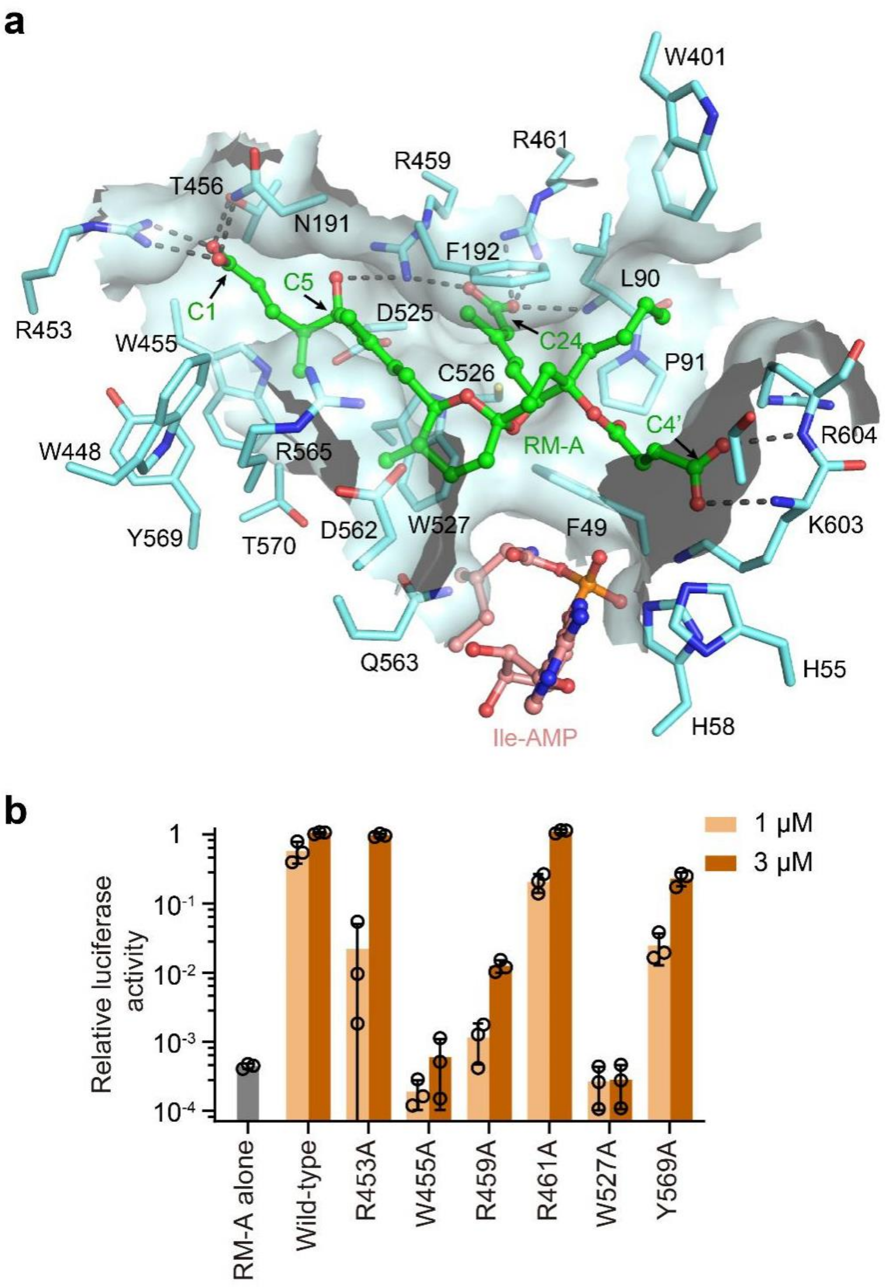

Supplementary Figure 8 | RM-A blocks human IleRS. a, The human IleRS structure was generated by the protein structure homology-modelling program of MOE. RM-A and Ile-AMP molecules were docked into the human IleRS active site in the program of MOE. **b**, C-terminal truncated human IleRS or its variants at 1 µM or 3 µM were added to rabbit reticulocyte lysate to test their capability to rescue the protein translation inhibited by 200 nM RM-A. All mutations largely reduced the rescue capability of human IleRS. Data are shown as mean ± SD (n = 3 independent experiments). Source data are provided as a Source Data file.

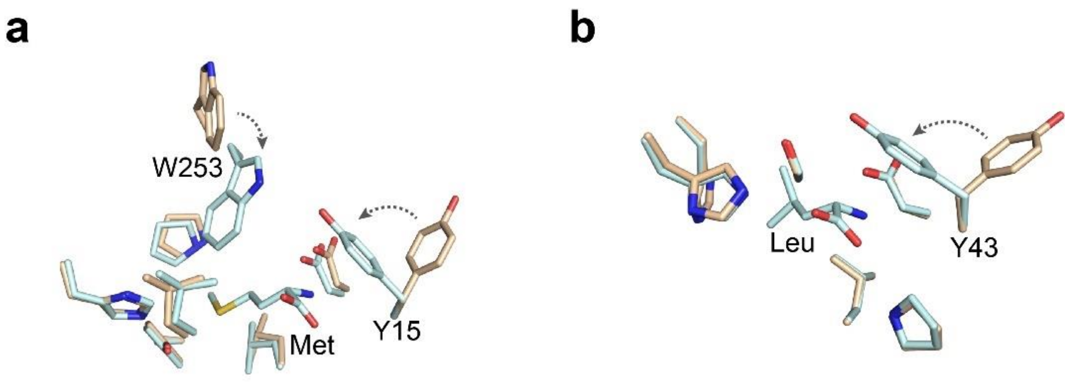

Supplementary Figure 9 | The flips of the aromatic residues in response to the binding of amino acids in *E. coil* MetRS and *E. coil* LeuRS. a, Close-up view of the amino acid binding sites of the apo MetRS (wheat, PDB ID: 1QQT) [http://doi.org/10.2210/pdb1QQT/pdb] and MetRS·L-methionine complex (cyan, PDB: 1F4L) [http://doi.org/10.2210/pdb1F4L/pdb]. **b**, Close-up view of the amino acid binding site of LeuRS with (cyan, PDB ID: 4ARC) [http://doi.org/10.2210/pdb4ARC/pdb] or without (wheat, PDB ID: 4ARI) [http://doi.org/10.2210/pdb4ARI/pdb] substrate L-leucine.

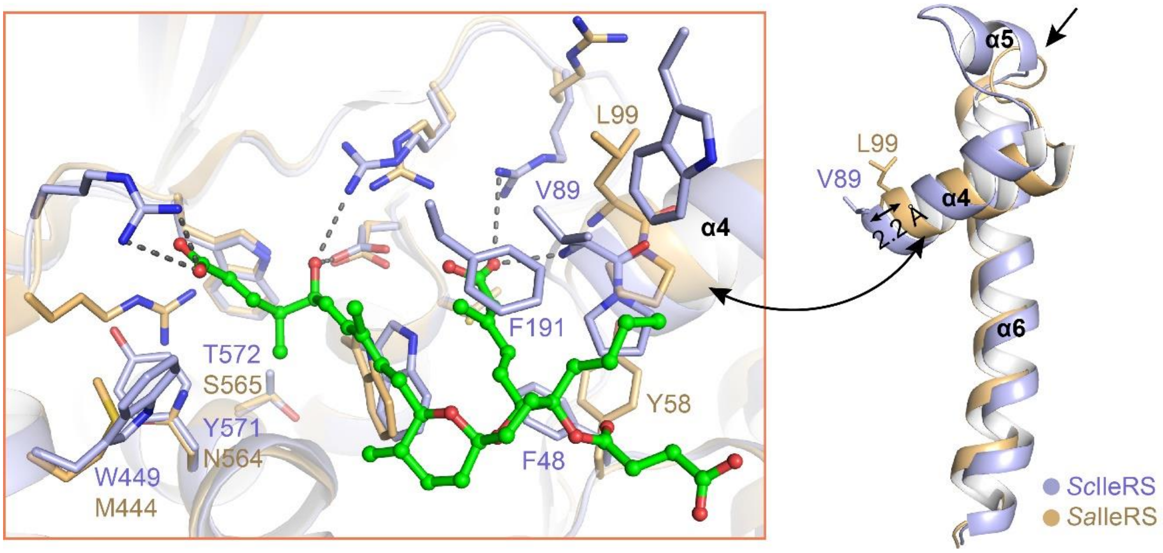

Supplementary Figure 10 | The structural insights into the insensitivity of bacterial IleRS to RM-A. Modelling RM-A in the aminoacylation pocket of *Sa*IleRS (wheat, PDB ID: 1QU2) [http://doi.org/10.2210/pdb1QU2/pdb] according to the *Sc*IleRS·RM-A·Ile-AMP complex structure. The residues forming hydrophobic or polar interactions with the C1-10 and C20-24 moieties of RM-A in *Sc*IleRS are partially substituted or conformation changed in *Sa*IleRS, which are possibly in charge of the insensitivity of bacterial IleRS to RM-A. The secondary structures are numbered according to *Sc*IleRS.

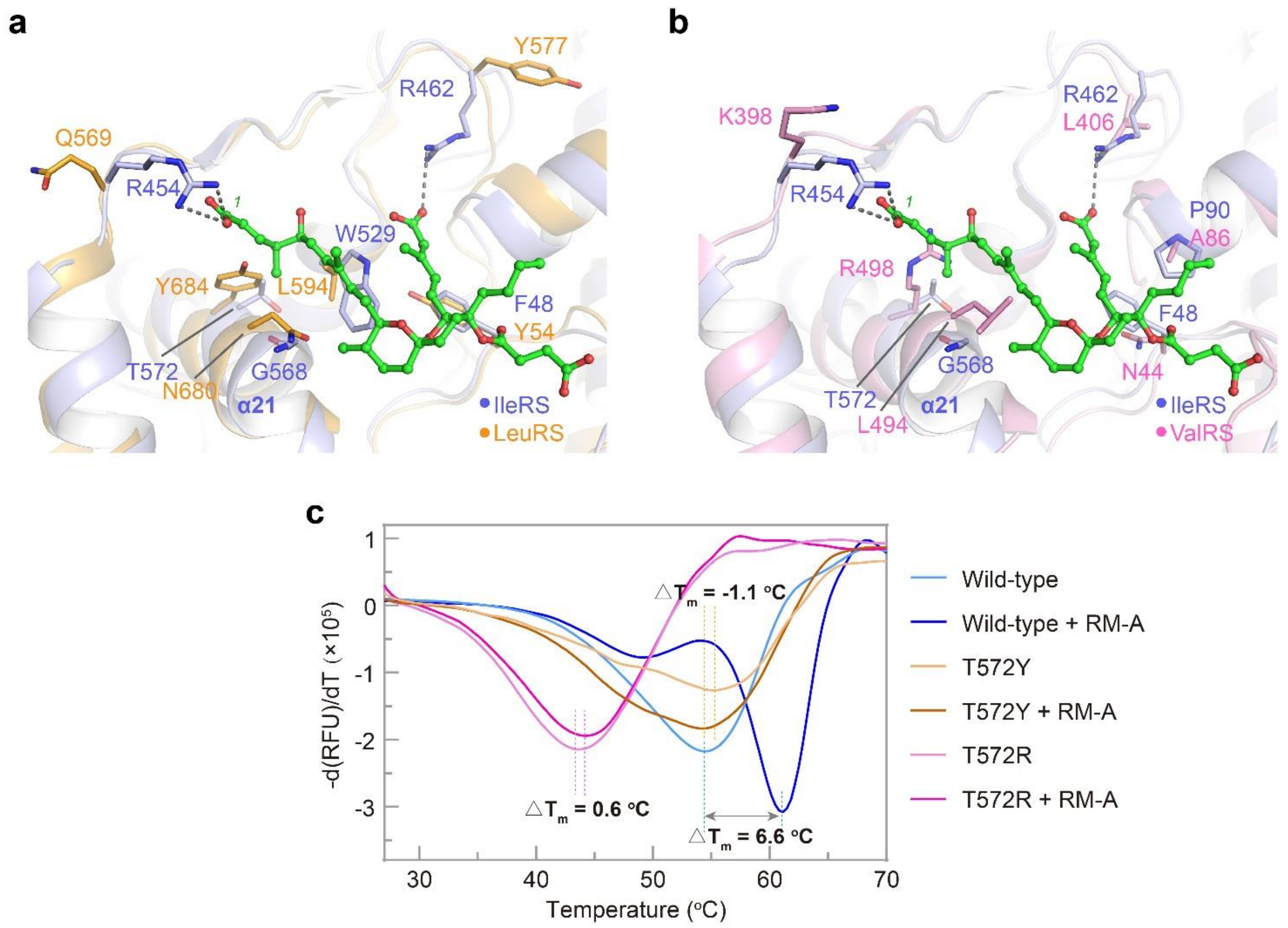

Supplementary Figure 11 | The possible structural explanations for the insensitivity of ValRS and LeuRS to RM-A. a-b, Modelling RM-A into LeuRS (**a**) and ValRS (**b**) by superposing the structure of the catalytic domain of *Sc*IleRS·RM-A·Ile-AMP complex (light blue) to that of human LeuRS (orange, PDB ID: 6LPF) [http://doi.org/10.2210/pdb6LPF/pdb] and *T. thermophilus* ValRS (pink, PDB ID: 1GAX) [http://doi.org/10.2210/pdb1GAX/pdb]. Many RM-A binding residues of *Sc*IleRS were found to be substituted to other residues in LeuRS and ValRS, which may miss some key interactions and also cause potential clashes between LeuRS/ValRS and modelled RM-A. **c,** Thermal melting curves of wild-type, T572R and T572Y *Sc*IleRS with or without RM-A. The effect of RM-A to the T_m value of each protein was labelled. The results showed that the T572R and T572Y variants of *Sc*IleRS lost the capability for binding RM-A.

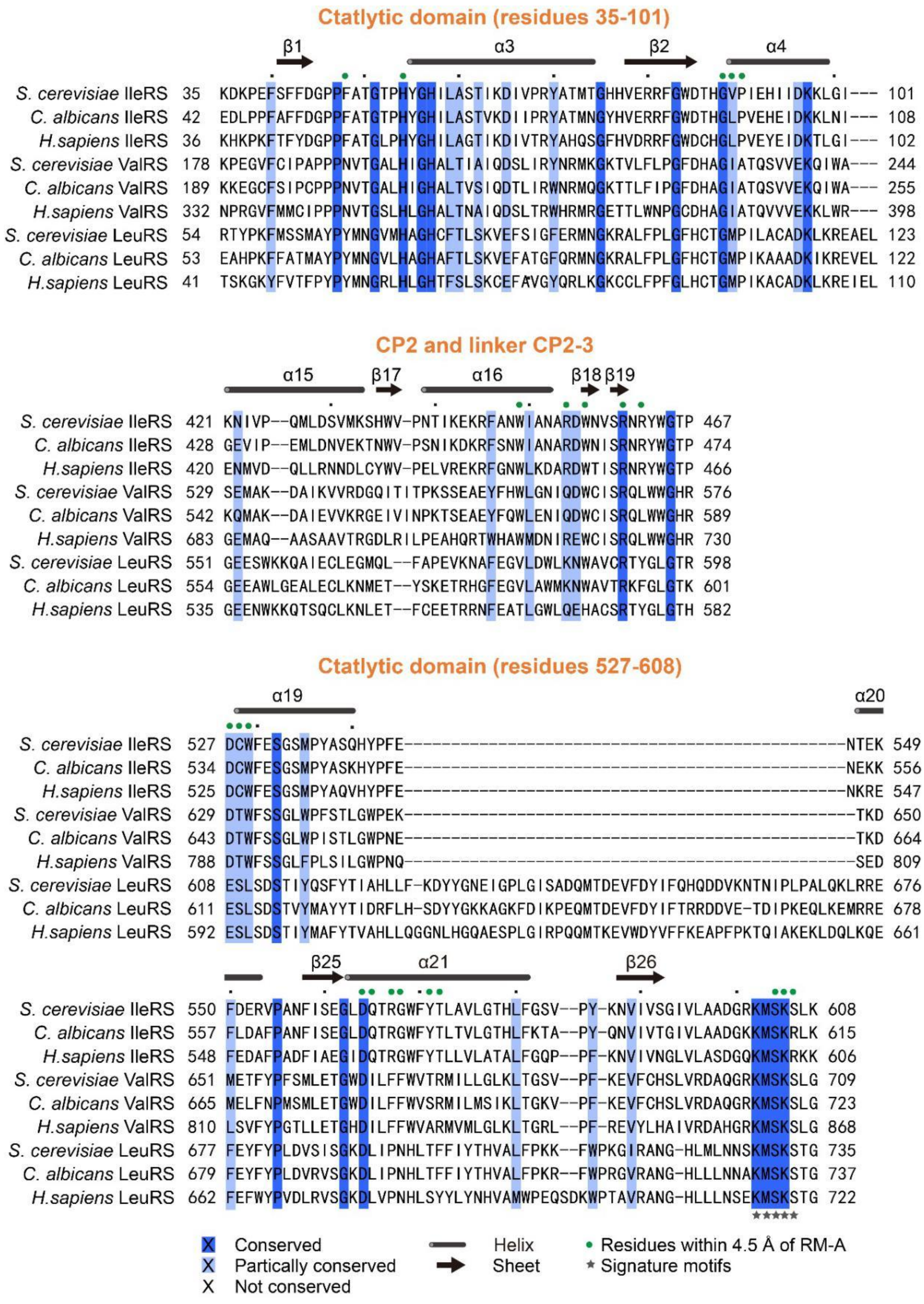

Supplementary Figure 12 | Structure-based sequence alignments of eukaryotic cytoplasmic IleRS, ValRS and LeuRS. The protein sequences of cytoplasmic IleRS, ValRS and LeuRS from *S. cerevisiae*, *C. albicans* and *H. sapiens* were aligned using Clustal Omega program[3] and then manually adjusted. The secondary structures corresponding to *Sc*IleRS are displayed above the sequences.

Supplementary Table 1 | Statistics of X-ray diffraction data collection and structure refinement.

	***Sc*IleRS·RM-A·Ile-AMP**
**Data collection**	
Resolution (Å)	50.00-1.90 (1.97-1.90)[a]
Space group	C121
Cell dimensions	
a, *b*, *c* (Å)	170.65, 64.76, 142.23
α, β, γ (°)	90.00, 107.78, 90.00
Unique reflections	117006 (11580)
R_{merge} (%)	9.5 (59.4)
Average $I/\sigma(I)$	16.5 (2.1)
Completeness (%)	99.9 (99.8)
Redundancy	3.3 (3.3)
**Refinement**	
Resolution (Å)	50.00-1.90 (1.95-1.90)
Reflections for refinement/test	111171/5835
R_{work}/R_{free}	0.177/0.194
No. atoms	
Protein	7510
Ligand	90
Water	673
B-factor (Å^2)	
Protein	21.03
Ligand	20.22
Water	30.66
R.m.s. deviations	
Bond lengths (Å)	0.005
Bond angles (°)	1.23
Ramachandran plot (%)	
Favored	97.3
Allowed	2.6
Outliers	0.1

[a]Values in parentheses are for the highest resolution shell.

Supplementary Table 2 | Summary of TSA and ITC results.

	△T_m[a] (°C)					K_d
	RM-A	Ile	RM-A + Ile	Ile-AMS	RM-A + Ile-AMS	
Wild-type	7	7.9	12.7	23.4	29.5	226 ± 17 nM
W449A	8.5	7.8	14.1	24.9	30.3	343 ± 63 nM
R454A	5	6.6	10.6	21.6	26.1	499 ± 57 nM
W456A	0.1	0.7	4.9	18.8	19.2	No binding
R460A	1.3	3.1	12	25.7	27.7	14.1 ± 3.5 μM
D527A	4.1	5.2	15.1	24.2	30.2	1.6 ± 0.2 μM
Y571A	2.4	4	13.3	24.6	26.1	3.2 ± 0.5 μM
T572A	5.5	4.8	11.9	22.7	27.2	340 ± 45 nM
W529A	0.1	1.9	2.2	18.4	18.5	No binding
R462A	3.7	4	12.3	26	28.8	2.4 ± 0.3 μM
F48A	1.9	0.2	2.6	12.5	12.9	7.2 ± 1.3 μM
F48Y	2	4.4	9.9	21.6	24.2	3.9 ± 0.7 μM
V89A	5.7	4.7	11.9	24.7	30	238 ± 36 nM
P90A	1.6	6.4	7.1	22.1	23.2	2.3 ± 0.3 μM
F191A	7.8	8.3	14.9	26.5	32	409 ± 83 nM
W402A	5.4	6.9	12.9	24.2	30.5	473 ± 48 nM
del_KMSKS	6.1	8.5	12.3	20.2	21.1	381 ± 28 nM

[a]△T_m is the difference between the T_m values of *Sc*IleRS variants with and without ligands.

Supplementary Table 3 | Primers used to construct recombinant plasmids.

Primer Name	Sequence
*Sc*IleRS	
FL/del_C -F	GGAATTCCATATGATGTCCGAGAGTAACGCACACTTCTC
FL -R	CCGCTCGAGTAATTTGAACACTTTTAATTTGAAAATGGTGTC
del_C -R	CCGCTCGAGACTCTTTAGTTCAGAGTAAATATTTGTATCCATG
W449A -F	CCAACGCTATCGCCAATGCCCGTGACTGGAACGT
W449A -R	GCGATAGCGTTGGCGAACCTCTTTTCCTTGATGGTGTTAG
R454A -F	ATGCCGCTGACTGGAACGTTTCCAGAAATAGATATTGG
R454A -R	CAGTCAGCGGCATTGGCGATCCAGTTGGCGAACCTC
W456A -F	GTGACGCTAACGTTTCCAGAAATAGATATTGGGGTACTCCAATTCC
W456A -R	ACGTTAGCGTCACGGGCATTGGCGATCCAGTTG
R460A -F	TTTCCGCTAATAGATATTGGGGTACTCCAATTCCTTTATGGGTTTCAG
R460A -R	CTATTAGCGGAAACGTTCCAGTCACGGGCATTGG
D527A -F	TTTTTGCTTGTTGGTTTGAATCTGGTTCTATGCCTTATGCTTCTC
D527A -R	CAACAAGCAAAAACTTCTTCAATTCTCTTTAAGTCACCCTTACCTTGCTTG
Y571A -F	GGTTCGCTACGTTAGCTGTCTTAGGTACCCATCTATTTGGCT
Y571A -R	CTAACGTAGCGAACCAACCTCTTGTTTGATCTAAACCTTCAGAGATGAAATTAGC
T572A -F	TCTACGCTTTAGCTGTCTTAGGTACCCATCTATTTGGCTCTGTTC
T572A -R	GCTAAAGCGTAGAACCAACCTCTTGTTTGATCTAAACCTTCAGAGATG
W529A -F	ATTGTGCTTTTGAATCTGGTTCTATGCCTTATGCTTCTCAACATTATCC
W529A -R	TCAAAAGCACAATCAAAAACTTCTTCAATTCTCTTTAAGTCACCCTTACCTTG
R462A -F	GAAATGCTTATTGGGGTACTCCAATTCCTTTATGGGTTTCAGACG
R462A -R	CAATAAGCATTTCTGGAAACGTTCCAGTCACGGGCATTG
F48A -F	CTCCAGCTGCCACCGGTACTCCTCATTACGGTCA
F48A -R	GTGGCAGCTGGAGGCCCATCGAAGAAGGAAAACTCC
F48Y -F	CTCCATACGCCACCGGTACTCCTCATTACGGTC
F48Y -R	GTGGCGTATGGAGGCCCATCGAAGAAGGAAAACTC
V89A -F	ACGGTGCTCCAATTGAACATATCATTGACAAGAAATTAGGTATCACGGGT
V89A -R	ATTGGAGCACCGTGTGTATCCCAACCGAATCTTCTTTCC
P90A -F	GTGTTGCTATTGAACATATCATTGACAAGAAATTAGGTATCACGGGTAAAGATGATG
P90A -R	TCAATAGCAACACCGTGTGTATCCCAACCGAATCTTC
F191A -F	GTAACGCTGAAGCTCAGCAAAACTATAAAGATGTC
F191A -R	GCTTCAGCGTTACTTAAGGGAGTGGTTAGCCCA
W402A -F	TCTGTGCTAGATCCGATACCCCATTGTTATACCGTTCTGTTC
W402A -R	GATCTAGCACAGAATGGATAGGAATGGCGAATTTGGGATG
del_KMSK -F	GATGGTTCCGGCTCTAATTACCCTGATCCATCCATTGTTCTGAACAAATATGGTG
del_KMSK -R	AGAGCCGGAACCATCGGCAGCTAAGACAATACCAGAGACGATG
human IleRS	
del_C -F	GGAATTCCATATGCTTCAACAAGTTCCAGAAAACATAAATTTTCCTGC
del_C -R	CCGCTCGAGATCTACCATTGACTGGTCAGGAGTGAC
R453A -F	ATGCAGCTGACTGGACAATTTCCAGAAACAGATACTGGGG
R453A -R	CAGTCAGCTGCATCTTTCAGCCAATTTCCAAATCGTTTTTCTC
W455A -F	GTGACGCTACAATTTCCAGAAACAGATACTGGGGCACC
W455A -R	ATTGTAGCGTCACGTGCATCTTTCAGCCAATTTCCAAATC
R459A -F	TTTCCGCTAACAGATACTGGGGCACCCCCATCC
R459A -R	CTGTTAGCGGAAATTGTCCAGTCACGTGCATCTTTCAG
R461A -F	GAAACGCTTACTGGGGCACCCCCATCCCACTGT
R461A -R	CAGTAAGCGTTTCTGGAAATTGTCCAGTCACGTGCATC
W527A -F	ACTGTGCTTTTGAGAGTGGCAGCATGCCCTATGCT
W527A -R	TCAAAAGCACAGTCAAACACTTCAGAGATGCGGTGC
Y569A-F	GGTTTGCTACCCTGCTGGTGCTGGCCACGGCCCT
Y569A -R	AGGGTAGCAAACCATCCTCTGGTTTGGTCGATGCCCTCG

Supplementary Table 4 | Primer sequences for preparation of DNA templates for in vitro transcription of tRNA^{Ile} and tRNA^{Pro}

tRNA^{Ile}	primer-I1	TAATACGACTCACTATAGGGCTTGTAGCTCAGGTG**GTTAGAGCGCACCCCTGATAAG**
	primer-I2	TGGTGGGCCTGAGTGGACTTGAACCACCGACCTCACC**CTTATCAGGGGTGCGCTCTAAC**
	primer-I3	TAATACGACTCACTATAGGGCTTGT
	primer-I4	TGGTGGGCCTGAGTGGACTTGAAC
tRNA^{Pro}	primer-P1	TAATACGACTCACTATAGGCGAGTAGCGCAGCTTG**GTAGCGCAACTGGTTTGGGACCA**
	primer-P2	TGGTCGGCGAGAGAGGATTCGAACCTCCGACCCAC**TGGTCCCAAACCAGTTGCGCTAC**
	primer-P3	TAATACGACTCACTATAGGCGAGTAGC
	primer-P4	TGGTCGGCGAGAGAGGATTCGAAC

Note: The bolded bases are the complementary sequences shared by the two primers (primer-I1 and -I2 for tRNA^{Ile}, and primer-P1 and -P2 for tRNA^{Pro}) used in the first PCR to obtain the double-stranded full-length DNA templates. The DNA templates were amplified in the second PCR using primer-I3 and -I4 for tRNA^{Ile} and primer-P3 and -P4 for tRNA^{Pro}.

Supplementary References

1. Shimizu, T. *et al*. Synthesis and biological activities of reveromycin A and spirofungin A derivatives. *Bioorg. Med. Chem. Lett.* **18,** 3756-3760 (2008).
2. Takahashi, S. *et al*. Structure-function analyses of cytochrome P450revI involved in reveromycin A biosynthesis and evaluation of the biological activity of its substrate, reveromycin T. *J. Biol. Chem.* **289,** 32446-32458 (2014).
3. Madeira, F. *et al*. The EMBL-EBI search and sequence analysis tools APIs in 2019. *Nucleic Acids Res.* **47,** W636-W641 (2019).
